# Evaluation of positioning accuracy, radiation dose and image quality: artificial intelligence based automatic versus manual positioning for CT KUB

**DOI:** 10.12688/f1000research.150779.1

**Published:** 2024-06-24

**Authors:** Souradip Kundu, Kaushik Nayak, Rajagopal Kadavigere, Saikiran Pendem, Priyanka

**Affiliations:** 1 Department of Medical Imaging Technology, Manipal College of Health Professions, Manipal Academy of Higher Education, Manipal, Karnataka, 576104, India; 2 Department of Radio Diagnosis and Imaging, Kasturba Medical College, Manipal Academy of Higher Education, Manipal, Karnataka, 576104, India

**Keywords:** Artificial Intelligence, Computed Tomography, Image quality, Positioning accuracy, Radiation dose

## Abstract

**Background:**

Recent innovations are making radiology more advanced for patient and patient services. Under the immense burden of radiology practice, Artificial Intelligence (AI) assists in obtaining Computed Tomography (CT) images with less scan time, proper patient placement, low radiation dose (RD), and improved image quality (IQ). Hence, the aim of this study was to evaluate and compare the positioning accuracy, RD, and IQ of AI-based automatic and manual positioning techniques for CT kidney ureters and bladder (CT KUB).

**Methods:**

This prospective study included 143 patients in each group who were referred for computed tomography (CT) KUB examination. Group 1 patients underwent manual positioning (MP), and group 2 patients underwent AI-based automatic positioning (AP) for CT KUB examination. The scanning protocol was kept constant for both the groups. The off-center distance, RD, and quantitative and qualitative IQ of each group were evaluated and compared.

**Results:**

The AP group (9.66±6.361 mm) had significantly less patient off-center distance than the MP group (15.12±9.55 mm). There was a significant reduction in RD in the AP group compared with that in the MP group. The quantitative image noise (IN) was lower, with a higher signal-to-noise ratio (SNR) and contrast-to-noise ratio (CNR) in the AP group than in the MP group (p<0.05). Qualitative IQ parameters such as IN, sharpness, and overall IQ also showed significant differences (p< 0.05), with higher scores in the AP group than in the MP group.

**Conclusions:**

The AI-based AP showed higher positioning accuracy with less off-center distance (44%), which resulted in 12% reduction in RD and improved IQ for CT KUB imaging compared with MP.

## Introduction

Computed Tomography (CT) is a valuable imaging modality for the diagnosis of various pathologies. However, CT scans use X-rays, which involve exposure to ionizing radiation. Therefore, the radiation exposure in CT must be kept “As Low as Reasonably Achievable (ALARA)”. ^1^^–^^3^ In recent years, several dose optimization tools have been introduced, such as “deep learning image reconstruction (DLIR)”, “iterative reconstruction (IR)”, “automatic tube current modulation (ATCM),” and “automatic tube voltage selection (ATVS)”. ^4^^–^^9^ In addition, proper patient positioning is crucial for obtaining higher image quality with an optimized radiation dose. ^10^

Radiology medical technologists can utilize laser lights to visually evaluate the patient’s central placement in CT imaging; however, this approach is user-dependent, and patient miscentering is common and well-documented problem that can have detrimental consequences. ^11^ If the patient is placed away from the gantry isocenter (i.e., the table is too up or down), the localizer image will be either enlarged or reduced in width. Furthermore, the use of ATCM along with patient miscentering could lead to an unacceptable Image quality (IQ) with an increase in the radiation dose (RD). ^12^^–^^14^ For manual positioning, the interaction between the radiographers and the patient poses a risk of cross-infection in patients with infectious diseases. ^14^

Recently, an artificial intelligence (AI)-based positioning camera, which works based on an AI algorithm that uses intelligence (a body contour detection algorithm) to detect a patient’s body using a three-dimensional (3D) camera. ^15^ Various companies have introduced AI-based contactless positions for patients. A 3D camera equipped with a visible light camera, an infrared light source, and a sensor was installed above the patient. It adjusts the height of the table and maintains the patient within the isocenter of the gantry. It also detects body contours and automatically positions the patient for CT examination according to the selected protocol. ^14^^–^^18^ Hence, this study aimed to evaluate and compare the positioning accuracy, IQ, and RD of AI-based automatic and manual positioning for CT Kidney Ureter and Bladder (KUB) imaging.

## Methods

### Study design

This is a prospective study. Ethical approval was obtained from the Institutional ethical committee (IEC 168/2023) of Kasturba Medical College and Hospital, Manipal, India on 7 ^th^ June 2023, and then the study was registered on Clinical Trial Registry- India (CTRI/2023/06/054173) on 20 ^th^ June 2023. Written informed consent was obtained from all participants for publication and participation in the data collection for the study.

### Eligibility criteria

The study included a total of 286 patients, with 143 patients in each group referred for CT KUB Imaging for various clinical indications such as evaluation of renal calculi, flank pain, kidney masses, and traumatic injury to the kidneys. Patients who were uncooperative and those with CT KUB images with artifacts (movement and metal) were excluded. Patient age and BMI were noted, and only patients with normal BMI were included. All patients underwent CT using a Philips Incisive 128 Slice CT Scanner.

### Patient positioning

Patients in group 1 Manual positioning (MP group) underwent CT KUB imaging using manual positioning. The patient was positioned supine on a scan table with the feet first towards the gantry, and the arms were extended and supported above the head. The table height was adjusted by the gantry-mounted adjustment button such that the horizontal laser beam coincided with the mid-coronal plane of the patient and to the gantry isocenter by visual inspection. The area covered the dome of the diaphragm immediately below the symphysis pubis.

Group 2 Automatic Positioning (AP group) patients underwent CT KUB imaging by AI-based automatic patient positioning, which included an AI-enabled camera mounted on the ceiling above the patient table.

An AI-based camera automatically detected the patient’s orientation in the supine position with the feet first into the gantry. After selecting the CT KUB protocol, the area of interest (from the diaphragm to the symphysis pubis) to be scanned was automatically detected using an AI – based camera, and the table height was adjusted to the gantry isocenter.

### Image acquisition

The image acquisition parameters were kept the same for both groups such as use of ATCM, tube voltage of 120 kVp, rotation time of 0.75s, pitch 1.0, matrix 512*512, slice thickness and increment 3 mm. The MP group patient images was reconstructed with IR technique – iDose ^4^- level 4 (Philips Health Care ^®^^,TM^). The AP group patient images were reconstructed with DLIR technique (Precise Image; Philips Health Care ^®^^,TM^). The axial CT images from both the groups were reconstructed to extended field of view (FOV) of 500 mm.

### Off-center distance measurement

**Figure 1.**
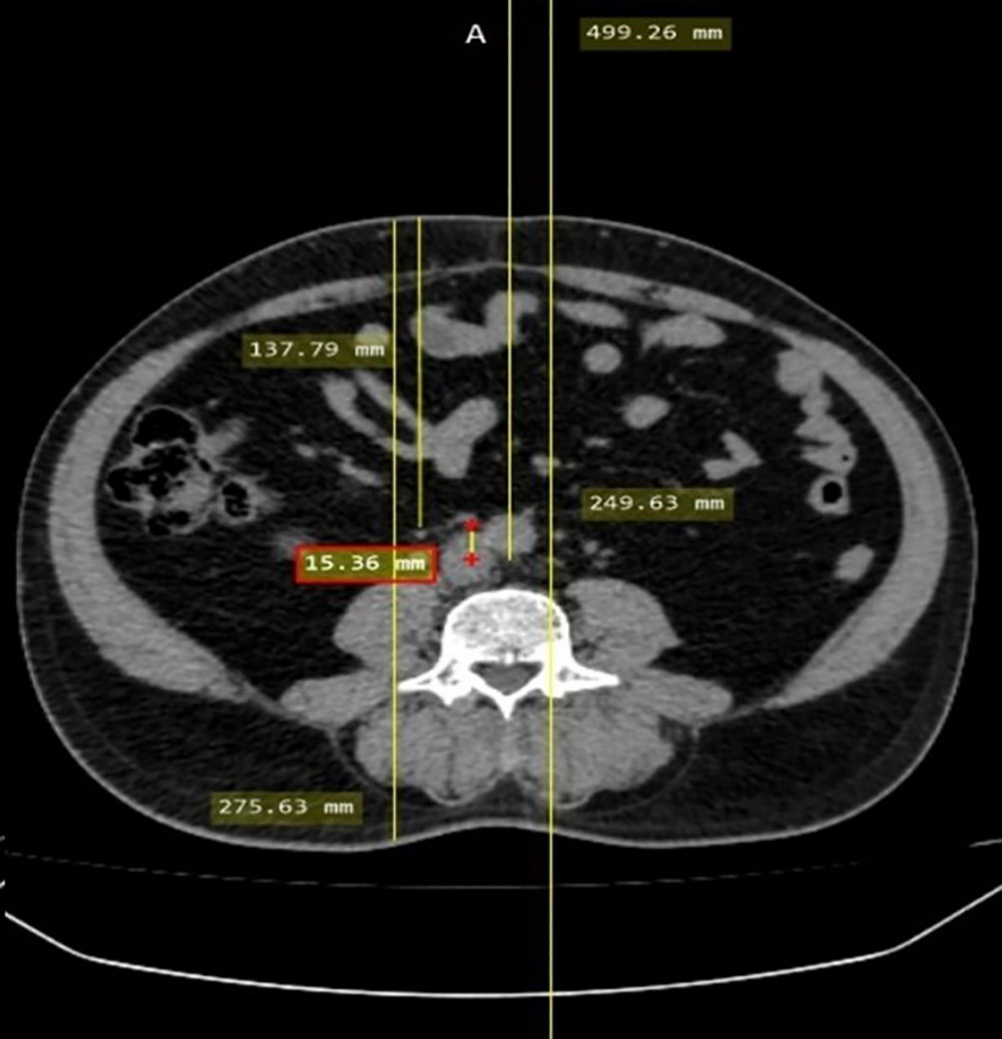
Off-center distance measurement.

The off-center distance was measured to evaluate the accuracy of patient positioning. To calculate the off-center distance, an axial slice of the CT KUB image at the level of the fourth lumbar vertebra, with an Field of View (FOV) of 500 mm, was selected. A straight line was drawn that joins the anterior and posterior margins of the complete FOV, and the midpoint of this line was determined, which represents the gantry isocenter. Another straight line that joined the anterior and posterior surfaces of the patient was drawn, and the midpoint of this line was determined to represent the patient’s center. The distance between the gantry isocenter and the patient’s center was measured using a measuring tool to evaluate the off-center distance ^12^ ( Figure 1). The scan length was noted in both the groups.

### Radiation dose measurement

Radiation dose descriptors such as “Volumetric Computed Tomography Dose Index (CTDIv) in mGy,” “Dose Length Product (DLP) in mGy.cm”, “Size Specific Dose Estimate (SSDE) in mGy” was noted from the CT scanner and the “effective dose (ED)” was calculated using the following formula: E=DLPXConversion factor(K) (K= 0.015 mSv/mGy. cm). ^19^

### Quantitative image quality

**Figure 2.**
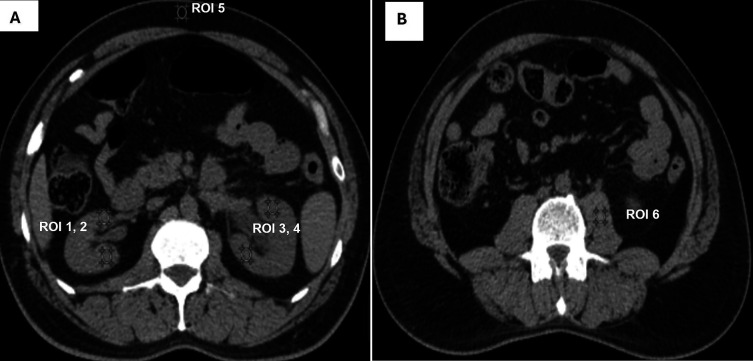
For quantitative assessment of image quality, A shows ROI were placed in upper (ROI 1) and lower pole (ROI 2) of right kidney, upper (ROI 3) and lower pole (ROI 4) of left kidney subcutaneous fat (ROI 5). B shows ROI placed in psoas muscle (ROI 6).

Quantitative IQ was assessed by calculating “signal to noise ratio (SNR),” “contrast to noise ratio (CNR)” and “image noise (IN).” 3 mm slice thickness axial CT KUB images were selected, and six circular regions of interest (ROI) measuring 4-5 mm ^2^ in diameter were drawn in the following regions: upper poles of the kidneys, lower poles, subcutaneous fat, and psoas muscle ( Figure 2A and 2B).

The IN was calculated as the standard deviation (SD) of the ROI, and the attenuation value was the mean value of the ROI. SNR and CNR were calculated using the following formula ^20^:

$$\mathrm{SNR}=\frac{\text{Attenuation value}}{\text{Image Noise}}$$

$$\mathrm{CNR}=\frac{\mathrm{CT}\;\text{Kidney attenuation value}–\text{CTPsoas muscle attenuation value}}{\text{Total image noise}}$$

### Qualitative analysis

**Figure 3.**
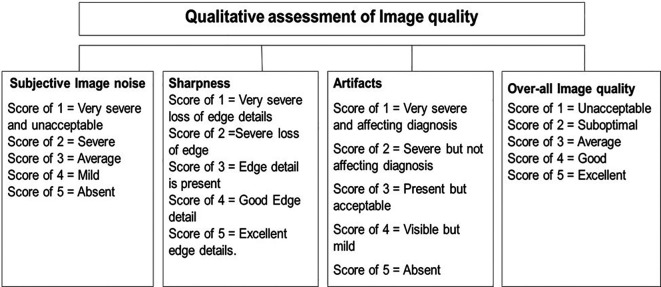
Qualitative analysis of image quality.

Qualitative analysis of IQ was performed by two radiologists with > 10 years of experience in CT KUB reporting who were blinded to the patient positioning and image reconstruction technique. They assessed “image noise (IN)”, “image sharpness (IS)”, “image artifacts (IA)”, and “overall image quality (OIQ)” using a five-point Likert scale, as shown in Figure 3.

### Statistical analysis

Statistical analysis of the data was performed using Jamovi -2.3.28.0 ( https://www.jamovi.org/download.html). An independent t-test was used to compare RD (CTDIvol, DLP, SSDE, and ED), scan length, quantitative IQ (attenuation, image noise, SNR, and CNR), and off-center distance between the MP and AP positioning groups. The Chi-square test was used to compare the qualitative analysis of IQ. Statistical significance was set at p< 0.05. Kappa value (k-value) was calculated to assess the interobserver agreement of qualitative IQ analysis and the scores as follows: almost perfect (0.81-0.99)”, “substantial (0.60 – 0.79)”, “moderate (0.40 – 0.59)”, “fair (0.21 – 0.39), “and “< 0.20, none to slight agreement”. ^21^

## Results

The study included 286 patients referred for CT KUB imaging, 143 patients underwent CT KUB imaging using manual positioning, and the remaining 143 patients underwent automatic positioning. Patient details are summarized in Table 1.

**Table 1.** Summary of patient details.

Demographic data	MP group	AP group
Gender, male: female	101:42	93:50
Age, mean (SD) (years)	46 (16.20)	44 (15.61)
Height, mean (SD) (cm)	168.7 (5.14)	170.14 (5.10)
Weight, mean (SD) (kg)	63.64 (5.69)	61.16 (6.09)
BMI, mean (SD)	22.36 (1.82)	21.12 (1.87)

MP Manual Positioning, AP Automatic Positioning, SD Standard Deviation, BMI Body Mass Index.

### Off-center distance

The mean off-center distance in the MP group and AP group was 15.12 ± 9.55 cm and 9.66 ± 6.361 cm. A statistically significant difference in the off-center distance (p < 0.05) was noted between the MP and AP group. The AP group showed 44% less off-center distance compared to the MP group. Scan length also showed a significant difference (p < 0.05) between the AP (56.0 ± 1.75 cm) and MP group (58.2 ± 3.55 cm).

### Radiation dose

The mean and standard deviation (SD) of the radiation dose indices for both groups are shown in Table 2. There was a statistically significant difference in the measured CTDIvol (p < 0.05), DLP (p < 0.05), SSDE (p < 0.05), and effective dose (p < 0.05) between the MP and AP groups. The AI based AP group showed 8.38%, 12.32%, 10.32%, 12.42% reductions in CTDIvol, DLP, SSDE, and ED, respectively, compared with MP group.

**Table 2.** Comparison of radiation dose indices between MP group and AP group.

Radiation dose Indices	MP group Mean (SD)	AP group Mean (SD)	p – value
CTDIvol (mGy)	8.207 (0.905)	7.541 (0.943)	<0.05
DLP (mGy.cm)	478.783 (67.111)	423.181 (63.529)	<0.05
SSDE (mGy)	10.081 (0.635)	9.092 (0.436)	<0.05
Effective dose (mSv)	7.182 (1.006)	6.348 (0.953)	<0.05

MP manual positioning, AP automatic positioning, CTDIvol volumetric computed tomography dose index, DLP dose length product, SSDE size-specific dose estimate, SD standard deviation, mGy milli-gray, mGycm milligray centimeter, mSv milli Sievert.

### Quantitative IQ

The mean and SD of the quantitative IQ parameters for both groups are shown in Table 3. Quantitative IQ parameters, such as attenuation of the right kidney (p = 0.740), left kidney (p = 0.570), psoas muscle (p = 0.157), and subcutaneous fat (p = 0.053), did not show significant differences between the MP and AP group. However, other parameters such as IN and SNR of the right kidney, left kidney, psoas muscle, and subcutaneous fat showed statistically significant differences (p < 0.05), with lower IN and higher SNR in the AP group than in the MP group. The AP group showed 46.42% total IN reduction compared to MP group Similarly, the CNR of right and left kidney was higher in AP group compared MP group with significant difference (p < 0.05).

**Table 3.** Quantitative IQ analysis between MP and AP group.

Quantitative IQ	MP group Mean (SD)	AP group Mean (SD)	p-value
**Attenuation (HU)**			
Right Kidney	29.059 (4.0)	28.83 (3.51)	0.740
Left Kidney	28.75 (3.90)	28.48 (3.39)	0.570
Psoas muscle	49.80 (5.33)	50.59 (5.37)	0.157
Subcutaneous fat	110.96 (7.68)	112.47 (6.16)	0.053
**Image noise (IN)**			
Right Kidney	13.35 (3.81)	8.03(1.97)	<0.05
Left Kidney	13.13 (3.75)	8.11(1.95)	<0.05
Psoas muscle	14.71 (3.93)	9.41 (2.19)	<0.05
Subcutaneous fat	10.26(3.32)	6.55 (2.33)	<0.05
Total IN	51.54 (11.02)	32.12 (6.44)	<0.05
**Signal to noise ratio (SNR)**			
Right Kidney	2.33 (0.71)	3.80 (1.03)	<0.05
Left Kidney	2.34 (0.70)	3.73(1.05)	<0.05
Psoas muscle	3.61 (1.01)	5.74 (1.81)	<0.05
Subcutaneous fat	12.31 (5.51)	19.08 (6.11)	<0.05
**Contrast to noise ratio (CNR)**			
Right Kidney	0.41 (0.14)	0.70 (08.24)	<0.05
Left Kidney	0.42 (0.14)	0.71 (0.25)	<0.05

MP Manual Positioning, AP Automatic Positioning, SD Standard Deviation, HU Hounsfield Unit.

### Qualitative IQ

**Figure 4.**
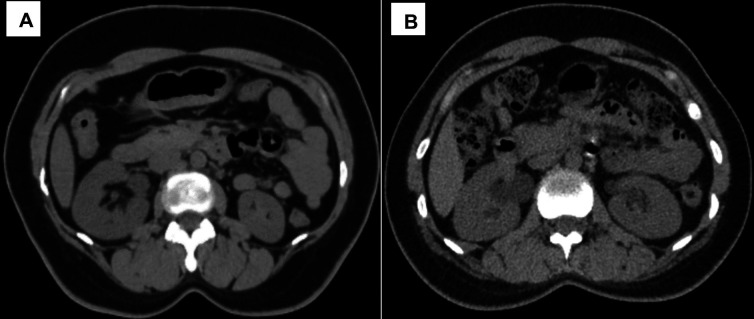
Axial CT KUB image acquired using AI-based automatic positioning technique (A). Axial CT KUB Image acquired using manual positioning technique (B).

The qualitative IQ scores of both readers in the MP and AP groups are shown in Table 4. IN, IS, and OIQ showed a statistically significant difference (p < 0.05) between the two groups, with higher scores in the AP group than in the MP group for both readers ( Figure 4). There was no significant difference in IA scores (p = 0.652) between the MP and AP group. However, none of the images were rated as suboptimal or unacceptable (score < 4) by the two readers. IN (MP, k = 0.98; AP, k = 0.88), IA (MP and AP, k = 1), IS (MP, k = 0.97; AP, k = 0.92), and OIQ (MP, k = 0.97; AP, k = 0.94) showed almost perfect inter-observer agreement between the two readers.

**Table 4.** Qualitative IQ analysis between Manual and Automatic positioning group.

Qualitative IQ	R1						R2						k-value	p-value (MP vs AP)	
Scores	1	2	3	4	5	Mean (SD)	1	2	3	4	5	Mean (SD)		R1	R2
**MP group**															
**IN**	0	0	0	128	15	4.10 (0.31)	0	0	0	127	16	4.11 (0.32)	0.98	<0.05	<0.05
**IA**	0	0	0	03	140	4.98 (0.15)	0	0	0	03	140	4.98 (0.15)	1.00	0.652	0.652
**IS**	0	0	0	20	123	4.86 (0.34)	0	0	0	20	123	4.86 (0.35)	0.97	<0.05	<0.05
**OIQ**	0	0	0	132	11	4.08 (0.26)	0	0	0	131	12	4.08 (0.28)	0.97	<0.05	<0.05
**AP group**															
**IN**	0	0	0	03	140	4.98 (0.15)	0	0	0	02	141	4.99 (0.12)	0.88	-	-
**IA**	0	0	0	02	141	4.99 (0.12)	0	0	0	02	141	4.99 (0.12)	1.00		
**IS**	0	0	0	04	139	4.97 (0.17)	0	0	0	01	142	4.98 (0.15)	0.92		
**OIQ**	0	0	0	05	138	4.97 (0.19)	0	0	0	04	139	4.97 (0.17)	0.94		

IQ image quality, IN image noise, IA image artifacts, IS image sharpness, OIQ overall image quality, MP manual positioning, AP automatic positioning, R1 Reader1, R2 reader 2, SD standard deviation.

## Discussion

In this study, we evaluated the positioning accuracy, IQ, and RD of artificial intelligence (AI)-based automatic and manual positioning for CT Kidney Ureter Bladder (KUB) imaging. A closer look at off-center distances showed that the off-center positions were significantly lower in the AI-based AP group than in the MP group. The mean off-center distances for the AP and MP groups were 9.662±6.36 mm. and 15.117±9.55 mm. Similar findings were observed in a study by Yadong et al., in which the off-center distance was significantly higher in the MP group (4.05 ± 2.40 cm) than in the AP group (1.56 ± 0.83 cm) for CT thorax imaging. ^14^ The study was performed by Ronald et al. on pediatric patients with and without immobilization devices. They found that utilizing the 3D camera for positioning pediatric patients, without an additional immobilization device, resulted in more precise positioning compared to manual methods employed by radiographers, which is similar to the findings of adults. Notably, there was no difference in the positioning accuracy between the 3D camera and radiographers for patients placed with an immobilization device. ^15^ Saltybaeva et al. evaluated the accuracy of the 3D camera algorithm for AP and compared the results with those of MP for both chest and abdominal CT. For chest CT, the average difference in off-center was 7 ± 4 mm when using AP and 19 ± 9 mm when the table height was selected manually by technologists. For the abdomen, the average vertical off-centering was 4 ± 2 mm and 18 ± 11 mm for the automatic and MP respectively. ^18^

AEC techniques are perhaps the most important innovations in terms of dose reduction. When using an AEC, the tube current is adjusted automatically based on the size and attenuation of the anatomy. ^22^^–^^24^ Off-center anatomy can result in suboptimal exposure settings, which affect IQ and increase RD. ^25^^,^^26^ In our study, there were notable reductions in radiation dose metrics such as CTDIv (8.38%), DLP (12.32%), ED (12.42%), and SSDE (10.32%) in AI-based AP group compared to MP group. Yadong et al. observed 16% dose reduction for AP in CT thorax examinations compared to MP. ^14^ Dane et al. showed 23.8%, 22.8%, 17.2 %, and 20.5 % reductions in radiation dose for CT chest without contrast, abdominal pelvis enterography, chest with contrast, and abdomen pelvis contrast studies, respectively, for 3D camera-based positioning. ^13^ Similar findings were noted in a study by Aly et al., who showed a higher off-center distance in the MP group than in the AP group. Due to the higher off-center distance, the radiation dose parameters such as CTDIv (abdomen: 10.2±4.3, 9.8 ±5.5 mGy; thorax: 8.6±4.3, 8.5 ±3.9 mGy), DLP (abdomen: 485.8±221.9, 492.2 ± 293.6 mGy.cm; thorax: 310.80±221.5, 319.0 ± 188.9 mGy.cm) and SSDE (abdomen: 13.1 ± 4.1, 12.9 ± 4.3 mGy; thorax: 10.5 ± 4.1, 10.7 ± 3.7 mGy) was higher in MP group compared to AP group. ^12^

The AI-based AP group CT images showed lower IN quantitatively and qualitatively than the MP group because of the higher positioning accuracy with less off-center distance in the AP group, and the images were reconstructed using the DLIR algorithm. SNR and CNR were also higher in the AP group than in the MP group. The qualitative IQ parameters, such as IS (MP: 4.86 ± 0.34; AP: 4.97 ± 0.17) and OIQ (MP: 4.08 ± 0.26, AP: 4.97 ± 0.19), showed higher mean scores in the AP group than in the MP group. Similar findings were reported by Yadong et al., in which the IN was lower in the AP group than in the MP group. ^14^^,^^27^

Our study had some limitations. First, we did not measure and compare patients positioning time in automatic and manual positions. Second, we could not assess whether the off-center distance observed in the study during manual patient positioning was below or above the gantry isocenter. Third, we enrolled patients with a normal BMI in this study. However, we did not specifically address how variations in patient weight may affect the accuracy of patient positioning.

## Conclusion

AI-based patient positioning is a touchless system that is operated by a single switch. The AI-based automatic positioning technique aligns the patient to the isocenter of the gantry with less off-center alignment and increases positioning accuracy. Hence, the study concludes that AI-based automatic positioning improves the overall image quality with noise reduction and reduced RD in patients undergoing CT KUB imaging. Further research in this area will improve the role of AI in healthcare optimization and patient care.

## Ethics and consent

Ethical approval was obtained from the Institutional ethical committee (IEC 168/2023) of Kasturba Medical College and Hospital, Manipal, India on 7 ^th^ June 2023, and then the study was registered on Clinical Trial Registry- India (CTRI/2023/06/054173) on 20 ^th^ June 2023. Written Informed consent was obtained from all participants for publication and participation in the data collection for the study.

## Data Availability

Figshare: AI based AP and MP CT KUB, https://doi.org/10.6084/m9.figshare.25641063.v3. ^28^ This project contains the following underlying data:
•
Data_AP_MP (demographic details of patients, Qualitative and Quantitative analysis, radiation dose metrics, and off-center distance – Excel sheet) • AP KUB Images, MP KUB Images Data are available under the terms of the Creative Commons Attribution 4.0 International license (CC-BY 4.0).
