# Supplementary material for: Evaluation of positioning accuracy, radiation dose and image quality: artificial intelligence based automatic versus manual positioning for CT KUB
Source: F1000Res. 2024 Jul 4;13:683. Originally published 2024 Jun 24. [Version 2] doi: 10.12688/f1000research.150779.2 (PMC11221346; doi:10.12688/f1000research.150779.2)
Supplement: Peer review history

# Reviewer report: Reviewer response for version 2

**DOI:** 10.5256/f1000research.168776.r296054

**Published:** 2024-07-09

**Authors:** Mustapha Barde

**Affiliations:** 1 Bayero University, Kano, Kano, Nigeria

The article presented a well sound approach in evaluating artificial intelligence (AI)-based method in comparison with manual patient positioning for CT KUB to ascertain the accuracy, radiation dose and image quality of the said methods. it was established that the AI based (automated position) is more robust Than the manual positioning, and it is recommended to be employed in clinical practice to optimize dose.

 The below minor corrections will be vital please.

 Minor correction 

1. The study duration (period within which it was conducted should be stated
2. It will be good to state that, manual approach was employed in off center distance measurement. Also, the lines indicating the off-center distance measurement (figure 1) should be labelled eg ( p,q, r…etc) to aid comprehension

Is the work clearly and accurately presented and does it cite the current literature?

Yes

If applicable, is the statistical analysis and its interpretation appropriate?

Yes

Are all the source data underlying the results available to ensure full reproducibility?

Yes

Is the study design appropriate and is the work technically sound?

Yes

Are the conclusions drawn adequately supported by the results?

Yes

Are sufficient details of methods and analysis provided to allow replication by others?

Yes

Reviewer Expertise:

Image analysis and processing, CT dosimetry

I confirm that I have read this submission and believe that I have an appropriate level of expertise to confirm that it is of an acceptable scientific standard.

# Reviewer report: Reviewer response for version 2

**DOI:** 10.5256/f1000research.168776.r299452

**Published:** 2024-07-09

**Authors:** Tasleem Shaikh

**Affiliations:** 1 Urgent Care, MedStar Health, Alexandria, Virginia, USA

The revised version is approved.

Is the work clearly and accurately presented and does it cite the current literature?

Yes

If applicable, is the statistical analysis and its interpretation appropriate?

Yes

Are all the source data underlying the results available to ensure full reproducibility?

Yes

Is the study design appropriate and is the work technically sound?

Yes

Are the conclusions drawn adequately supported by the results?

Yes

Are sufficient details of methods and analysis provided to allow replication by others?

Yes

Reviewer Expertise:

Medical Imaging Technology.

I confirm that I have read this submission and believe that I have an appropriate level of expertise to confirm that it is of an acceptable scientific standard.

# Reviewer report: Reviewer response for version 2

**DOI:** 10.5256/f1000research.168776.r299451

**Published:** 2024-07-05

**Authors:** Chandrasekhar Priyanka

**Affiliations:** 1 Apollo University, Chittoor, Andhra Pradesh, India

I approve this revised version.

Is the work clearly and accurately presented and does it cite the current literature?

Yes

If applicable, is the statistical analysis and its interpretation appropriate?

Yes

Are all the source data underlying the results available to ensure full reproducibility?

Yes

Is the study design appropriate and is the work technically sound?

Yes

Are the conclusions drawn adequately supported by the results?

Yes

Are sufficient details of methods and analysis provided to allow replication by others?

Yes

Reviewer Expertise:

Computed Tomography,Magnetic Resonance Imaging, Radiation dose, Mammography and Fetal MRI

I confirm that I have read this submission and believe that I have an appropriate level of expertise to confirm that it is of an acceptable scientific standard.

# Reviewer report: Reviewer response for version 1

**DOI:** 10.5256/f1000research.165377.r296053

**Published:** 2024-07-01

**Authors:** Tasleem Shaikh

**Affiliations:** 1 Urgent Care, MedStar Health, Alexandria, Virginia, USA

**Major comments:**

 The article highlights the advantages of AI-based automatic positioning using precise images in terms of positioning accuracy, radiation dose, and image quality compared to manual positioning for CT KUB examination.

 The introduction highlights the importance of reducing radiation dose in CT examinations using AI-based automatic positioning, which improves positioning accuracy and image quality with adherence to the ALARA principle.

 The methodology is well explained. Inclusion criteria for patient selection, measurement of radiation dose parameters, image quality (quantitative and qualitative) parameters, off-center distance, and standardized protocols for both AI-based and manual positioning enhance the study's reproducibility. Appropriate statistical methods were used to analyze the data.

 The results section presents the findings clearly and organized, using appropriate tables and figures to illustrate the data. The significant improvements in positioning accuracy and reduction in radiation dose with AI-based positioning are well-documented. The 44% reduction in off-center distance suggests a substantial improvement in patient positioning accuracy, which is essential for optimal imaging. The statistically significant reductions in CTDIvol (8.38%), DLP (12.32%), SSDE (10.32%), and ED (12.42%) for the AP group compared to the MP group are noted. Higher qualitative IQ scores support the benefits of AI-based positioning in producing better diagnostic image quality. The almost perfect interobserver agreement suggests that these results are reliable and reproducible.

 The discussion effectively summarizes the results and implications of improved positioning accuracy and reduced radiation dose for clinical practice. AI's potential to enhance consistency and efficiency in CT KUB scans is well-articulated, and the limitations of the study are well-addressed.

 The conclusion highlights the potential of AI-based automatic positioning to improve clinical outcomes and reduce radiation exposure for CT KUB examinations.

**Minor comments:**

 Ensure all the abbreviations are clearly defined when first used in the manuscript.

 Include a section on future research directions of the study. Suggest how the findings of this study could be applied or expanded in future research.

 Check for typographic errors in the units.

 The study is well structured, with clear comparisons and statistically significant results that highlight the potential of AI in CT imaging procedures, leading to better patient outcomes and improved workflow in radiology.

Is the work clearly and accurately presented and does it cite the current literature?

Yes

If applicable, is the statistical analysis and its interpretation appropriate?

Yes

Are all the source data underlying the results available to ensure full reproducibility?

Yes

Is the study design appropriate and is the work technically sound?

Yes

Are the conclusions drawn adequately supported by the results?

Yes

Are sufficient details of methods and analysis provided to allow replication by others?

Yes

Reviewer Expertise:

Medical Imaging Technology.

I confirm that I have read this submission and believe that I have an appropriate level of expertise to confirm that it is of an acceptable scientific standard.

# Reviewer report: Reviewer response for version 1

**DOI:** 10.5256/f1000research.165377.r296056

**Published:** 2024-07-01

**Authors:** Chandrasekhar Priyanka

**Affiliations:** 1 Apollo University, Chittoor, Andhra Pradesh, India

The article is interesting and innovative in exploring the advantages of AI-based positioning camera which detects the patients contour and allows for automatic positioning in CT KUB examination.

**Major comments**

 The article highlights the AI-based positioning ability of accurately positioning the patient with respect to the isocenter of gantry. It explains the benefits of reduced radiation dose by positioning the patient to isocenter in CT.

 The improvement in image quality with AI-based deep learning reconstruction algorithm (DLR) with automatic positioning such as precise image compared to iterative reconstruction (iDose ^4^) with manual positioning for CT KUB examinations was presented well in the article using qualitative and quantitative image quality analysis.

 Radiation is an important concern while performing CT scans. The present study also highlights the 12% reduction in radiation dose which is a substantial improvement, contributing to safer imaging practices.

**Minor comments:**

 A small typographic error was noted in the units of off-center distance between abstract (mm) and results section (cm) which can be corrected.

 The study focused on CT KUB, requires exploration in other body parts for comprehensive understanding and realizing the full potential of AI-based positioning techniques in CT.

 Overall, I feel the article is excellent in the current era of utilizing the advantages of artificial intelligence-based technologies for optimizing the workflow, improving the patient care and comfort in CT.

Is the work clearly and accurately presented and does it cite the current literature?

Yes

If applicable, is the statistical analysis and its interpretation appropriate?

Yes

Are all the source data underlying the results available to ensure full reproducibility?

Yes

Is the study design appropriate and is the work technically sound?

Yes

Are the conclusions drawn adequately supported by the results?

Yes

Are sufficient details of methods and analysis provided to allow replication by others?

Yes

Reviewer Expertise:

Computed Tomography,Magnetic Resonance Imaging, Radiation dose, Mammography and Fetal MRI

I confirm that I have read this submission and believe that I have an appropriate level of expertise to confirm that it is of an acceptable scientific standard.
